# TME12/400: Application Oriented Wavelet-based Coding of Volumetric Medical Data

**DOI:** 10.2196/jmir.1.suppl1.e119

**Published:** 1999-09-19

**Authors:** G Menegaz, L Grewe, A Lozano, J-Ph Thiran

**Affiliations:** ^1^LST-DE EPFL, LausanneSwitzerland

**Keywords:** Telemedicine, Compression

## Abstract

**Introduction:**

While medical data are increasingly acquired in a multidimensional space, in clinical practice they are mainly still analyzed as images. We propose a wavelet-based coding technique exploiting the full dimensionality of the data distribution while allowing to recover a single image without any need to decode the whole volume. The proposed compression scheme is based on the Layered Zero Coding (LZC) method. Two modes are considered. In the progressive (PROG) mode, the volume is processed as a whole, while in the layer-per-layer (LPL) one each layer of each sub-band is encoded independently. The three-dimensional extension of the Embedded Zerotree Wavelet (EZW) coder is used as reference for coding efficiency. All working modalities provide a fully embedded bit-stream allowing a progressive by quality recovering of the encoded information.

**Methods:**

The 3D DWT is performed mapping integers to integers thus allowing lossless compression. Two different coding systems have been considered: EZW and LZC. LZC models the expected statistical dependencies among coefficients by defining some conditional terms (contexts) which summarize the significance state of the samples belonging to a generalized neighborhood of the coefficient being encoded. Such terms are then used by a context adaptive arithmetic coder. The LPL mode has been designed in order to be able to independently decode any image of the dataset, and it is derived from the PROG mode by over-constraining the system. The sub-bands are quantized and encoded according to a sequence of uniform quantizers with decreasing step-size. This ensures progressiveness capabilities when decoding both the whole volume and a single image.

**Results:**

Performances have been evaluated on two datasets: DSR and ANGIO, an opthalmologic angiographic sequence. For each mode the best context has been retained. Results show that the proposed system is competitive with EZW, and PROG mode is the more performant. The main factors compromising compression efficiency in the LPL mode are the restriction on the choice of the contexts, and the overheading resulting from the independent coding of each layer in each sub-band. This mainly depends on the number of decomposition levels and the volume size. The isotropy of the data distribution for DSR volume results in better compression efficiency.

**Discussion:**

The exploitation of the whole 3D correlation among data samples improves coding efficiency with respect to 2D systems, encoding each image independently. The number of layers to be decoded in the LPL mode in each sub-band to recover a given image is a function of the length of Wavelet filters, which makes short ones particularly suited. For the filter used the maximum this number is 4. This makes the system particularly efficient at decoding, ensuring a fast and effective access to data. Among the possible applications, it is worth citing low-rate transmission (telemedicine), archiving and remote access.

